# Two new benthic *Euphilomedes* Kornicker, 1967 (Ostracoda, Myodocopida, Philomedidae) from the Taiwan Strait (East China Sea)

**DOI:** 10.7717/peerj.3146

**Published:** 2017-04-06

**Authors:** Peng Xiang, Xiaoyin Chen, Ruixiang Chen, Jinghong Lin, Yu Wang, Youyin Ye, Mao Lin

**Affiliations:** 1 Laboratory of Marine Biology and Ecology, Third Institute of Oceanography, State Ocean Administration, Xiamen, China; 2 Collaborative Innovation Center of Deep Sea Biology, Hangzhou, China

**Keywords:** Taxonomy, Ostracoda, *Euphilomedes*, Taiwan Strait, New species

## Abstract

Ostracods are small bivalved crustaceans widely distributed in aquatic environments and in total 99 species have been recorded in genus *Euphilomedes* Kornicker, 1967. In this study, we further describe two new species of benthic ostracods in this genus, *Euphilomedes liuruiyii* sp. nov. and *Euphilomedes pentacanthos* sp. nov., collected from the Taiwan Strait in China. These two species can be easily distinguished from their congeners by some crucial morphological characteristics, including the carapace shape, the numbers of main and secondary furcal claws, and their arrangement of furcal claws. In addition the first antenna, endopod of second antenna, frontal organ, mandible and the seventh limb also differentiate these two species from related species.

## Introduction

Ostracods are small bivalved crustaceans that inhabit various aquatic environments where they are one of the major constituents of the meiobenthos. The taxonomy and ecology of marine ostracods have been well investigated in the past two decades, but information on benthic ostracods remains limited in China.

The genus *Euphilomedes* Poulsen, 1962, belonging to subfamily Philomedinae Müller, 1908, family Philomedidae Müller, 1906, order Myodocopida Sars, 1866, was originally erected by Poulsen (1962), and has the second largest number of ostracod species following the genus *Philomedes* Lilljeborg, 1853 in the family (Brandão et al., 2016). Poulsen (1962) proposed the diagnosis of the genus and concluded there were nineteen species within the genus, and Kornicker (1967) further designated *Euphilomedes nodosus* Poulsen, 1962 as the type species of the genus.

The genus *Euphilomedes* has a worldwide distribution and typically inhabits marine epibenthic environments (Brady, 1897; Graf, 1931; Klie, 1940; Kornicker, 1967, 1974, 1995; Hartmann-Schröder & Hartmann, 1974; Cohen & Kornicker, 1975; Kornicker & Caraon, 1977; Chen, 1984; Kornicker & Harrison-Nelson, 1997; Chavtur et al., 2007). In total 29 *Euphilomedes* species have been reported in previous studies (Müller, 1890; Brady, 1890, 1902; Kajiyama, 1912; Poulsen, 1962; Hiruta, 1976; Hartmann, 1985; Kornicker, 1991; Chen et al., 2015a, 2015b; Brandão et al., 2016; detailed species is given in Table S1). Given their wide distribution and potentially high diversity, it is known that many as yet unnamed species await description (Karanovic, 2010). In this study, we describe two new species of genus *Euphilomedes* collected from the Taiwan Strait, China, based on the detailed structures of the carapaces and appendages.

## Materials and Methods

Collections were conducted in 1984–1985 in the coastal waters of the Taiwan Strait (from 0 to 200 m depths) during a comprehensive study on the Taiwan Strait. The study was sponsored by the State Oceanic Administration (SOA), China; there are no specific permissions required for the sampling activities in the coastal areas.

A standard plankton sampling net (net mouth diameter 80 cm, mesh aperture 0.505 mm) was used for a vertical drag from bottom to surface water. Collections were immediately fixed with 5% buffered formaldehyde for long-term preservation and sorted in the laboratory. The sub-samples of ostracods were isolated from the fixed collections, identified and dissected under a zoom-stereomicroscope (Zeiss Discovery V2.0).

Dissected appendages were mounted in permanent slides with CMC-9AF mounting medium (Masters Company Inc., Wood Dale, IL, USA). Observation was done using a transmitted-light binocular microscope combined with a differential interference contrast system and AxioVision Image-Pro software (Axio Imager Z2, Carl Zeiss Inc., Oberkochen, Germany). Drawings and measurements were done following those outlined in Karanovic (2010). All drawings were made from preserved specimens using a camera Lucida and a drawing apparatus, and further processed with Adobe Photoshop CS6 (Adobe Inc., San Jose, CA, USA).

Specimens of these two species were deposited in the Marine Biological Sample Museum of the Chinese Offshore Investigation and Assessment, the Third Institute of Oceanography, SOA, China (Xiamen, China), under the collection numbers TIO-OMPEu 301–303 and TIO-OMPEu 311–312 for *Euphilomedes liuruiyii* sp. nov. and *Euphilomedes pentacanthos* sp. nov., respectively.

### Nomenclatural acts

The electronic version of this article in portable document format will represent a published work according to the International Commission on Zoological Nomenclature (ICZN), and hence the new names contained in the electronic version are valid. This published work and the nomenclatural acts with this article contains have been registered in ZooBank. The LSID for this publication is: urn:lsid:zoobank.org:pub:9E34993C-1881-4D7F-919B-BCA046F411BB. The online version of this work is archived and available from the following digital repositories: PeerJ, PubMed Central, and CLOCKSS.

## Results

### Systematic account

**Figure 1:**
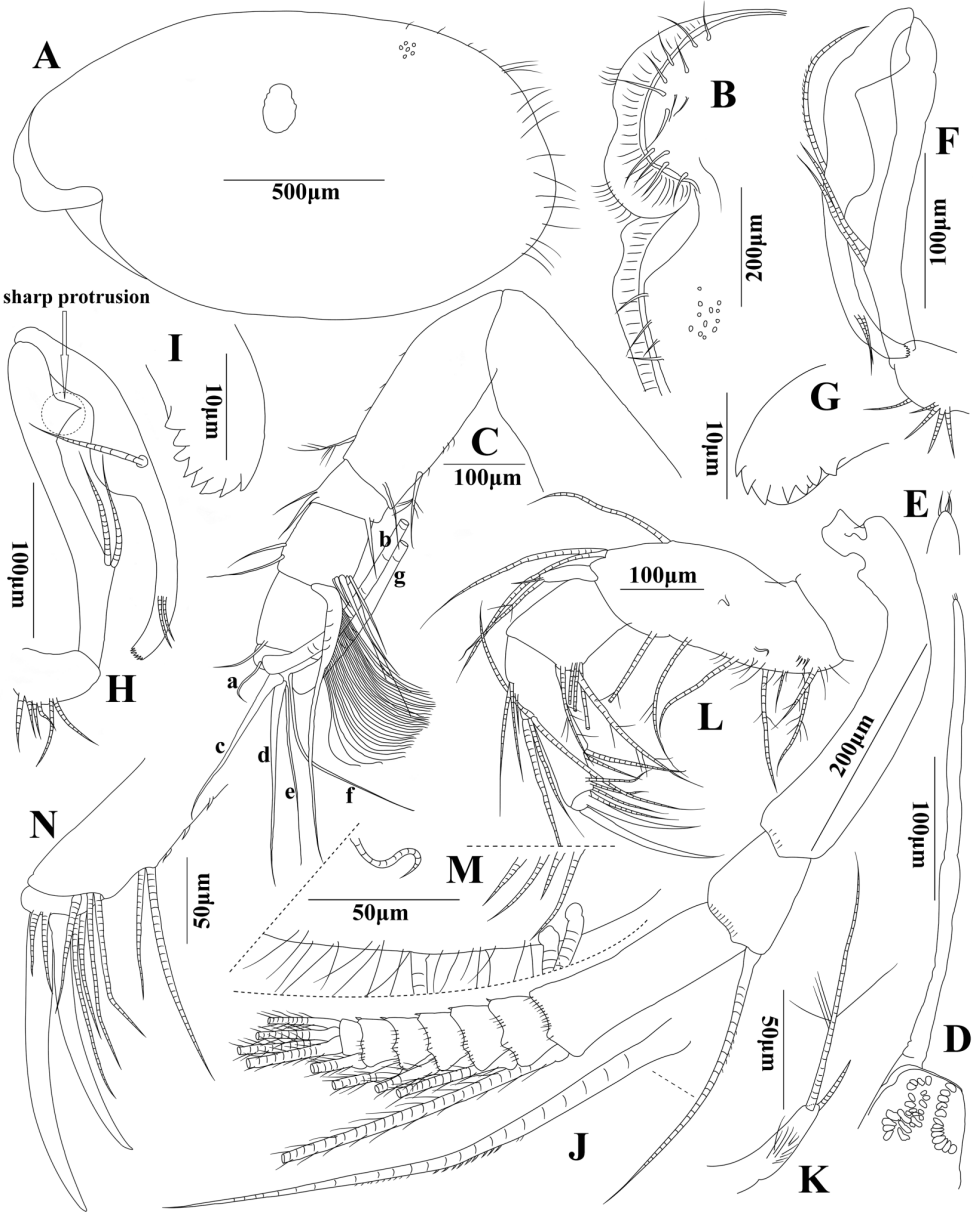
*Euphilomedes liuruiyii* sp. nov. (A) Left carapace, lateral view. (B) Rostrum of right carapace, medial view. (C) First antenna, lateral view. (D) Frontal organ, lateral view. (E) Tip of frontal organ, lateral view. (F) Left endopod of second antenna, lateral view. (G) Tip of endopod of left second antenna, lateral view. (H) Right endopod of second antenna, lateral view. (I) Tip of endopod of right second antenna. (J) Exopod of second antenna, lateral view. (K) Exopod of mandible, lateral view. (L) Mandible, lateral view. (M) Ventral margin of mandible, lateral view. (N) Tip of mandible, lateral view.

**Figure 2:**
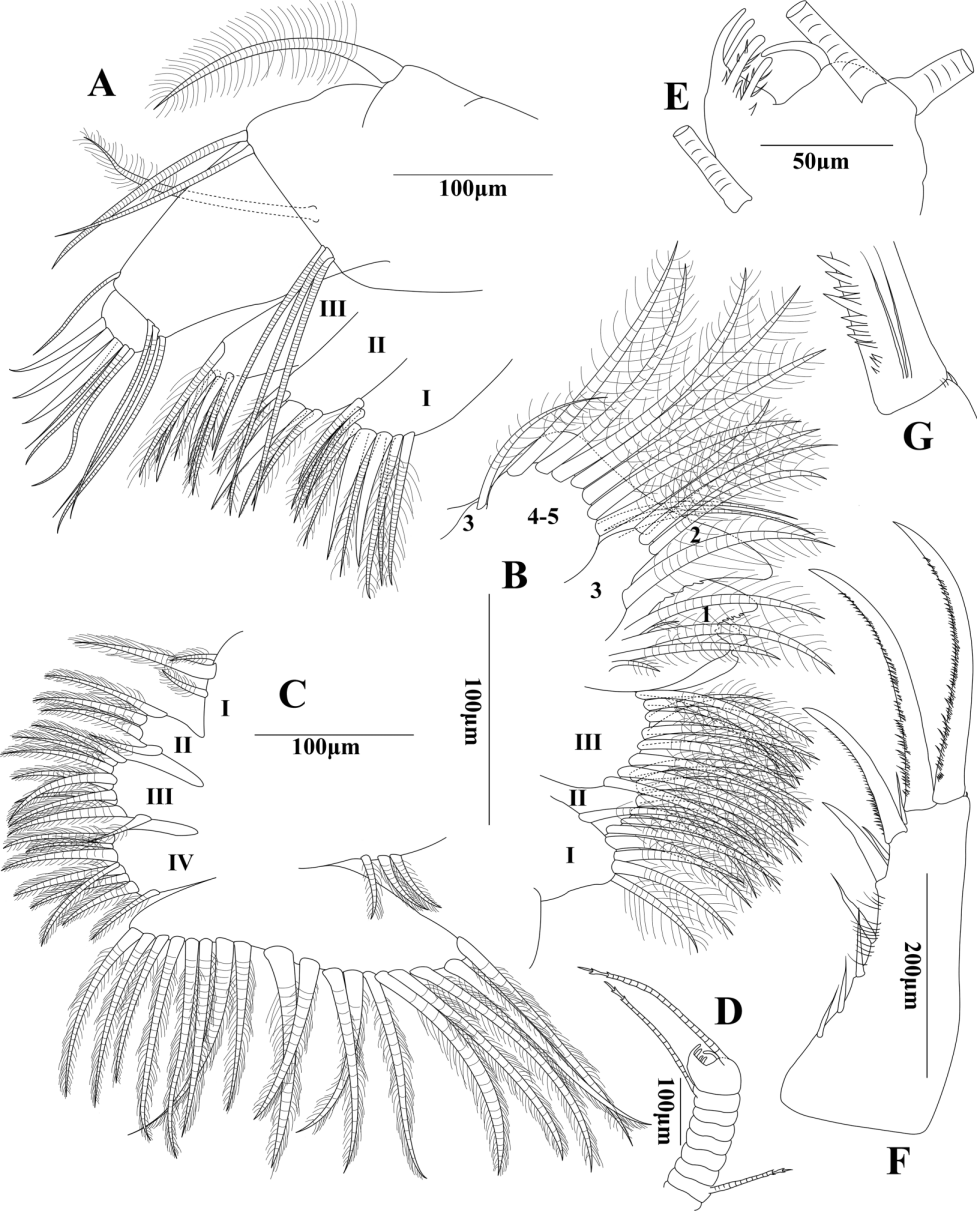
*Euphilomedes liuruiyii* sp. nov. (A) Maxilla, lateral view. (B) Fifth limb, lateral view. (C) Sixth limb, lateral view. (D) Seventh limb, lateral view. (E) Tip of seventh limb, lateral view. (F) Furca, lateral view. (G) Detail of disto-dorsal claw corner of furca, lateral view.

Order Myodocopida Sars, 1866
Family Philomedidae Müller, 1906
Genus *Euphilomedes* Poulsen, 1962
***Euphilomedes liuruiyii* sp. nov. Chen & Xiang**
urn:lsid:zoobank.org:act:51A26454-F244-4C42-921A-667846DF529D
Figures 1 and 2.

**Etymology:** The new species name is a Latinized name of Ruiyu Liu, an academician of the Chinese Academy of Sciences, in recognition of his important contributions to the development of marine biology of China.

**Holotype:** Collection number TIO-OMPEu 301, adult male, length 1.68 mm, height 0.97 mm, dissected on slides, carapace in alcohol. Type locality: offshore of southeast of Meizhou Island, the Taiwan Strait (25°01.00′N, 119°10.02′E; depth 21 m), Nov 12, 1984.

**Paratypes:** Collection number TIO-OMPEu 302, adult male, length 1.65 mm, height 0.96 mm, dissected on slide, carapace in alcohol; collection number TIO-OMPEu 303, length 1.60 mm, height 0.92 mm, deposited in 5% buffered formaldehyde. Paratypes collected in the same way as the holotype.

**Distribution:** Known at present only from type locality.

**Diagnosis:** Height about 58% of length. Carapace with projecting rostrum and shallow incisure, rostral infold depressed. Frontal organ long straight rod and with only one article with slightly inflated mid part, and three small spines on blunt tip. Basale of mandible with one short hook-shaped seta. Comb of seventh limb with four teeth, two inner comb teeth with two basal spines each, and side opposite comb with one bare bending peg. Each furcal lamella with 10 claws and one small spine on disto-dorsal claw corner, claws one, two, four, and six primary, claws three, five, and seven to 10 secondary.

### Description

**Carapace (Figs. 1A and 1B):** Ornamentation: carapace oval in lateral view with round and oval carapace pits on surface, long setae on posterior carapace, projecting rostrum and shallow incisure, rostral infold depressed with distal cilia and ventral cilia. Posterior margin arced with long spines, postero-ventral margin slightly hump shaped. Carapace length 1.60–1.68 mm, height 0.92–0.97 mm, height about 58% of length, greatest height near mid-part of carapace.

**First antenna (Fig. 1C):** First antenna with six articles. Article 1 bare and long. Article 2 long with short spines and three setae, one disto-ventral plumose seta, one disto-dorsal plumose seta and one long disto-medial seta. Article 3 very short, with one long spine on disto-ventral margin, and two long setae on disto-dorsal margin. Article 4 approximately two times length of article 3, with two long disto-dorsal setae and one cluster of unequal setae, one equilong to article 4 on disto-ventral margin, with about 30 very long soft ventral filaments and one long sharp distal seta approximately equilong to article 2. Article 5 equilong to article 4 with one disto-dorsal seta. Article 6 very short with seven sensory setae, a-setae short and bent, b- and g-setae very grand and long, c- and d-setae long and larger than e- and f-setae.

**Frontal organ (Figs. 2D and 2E):** Frontal organ long straight and rod, with only one article with slightly inflated mid part, and three small spines on blunt tip.

**Second antenna (Figs. 1F–1J):** Second antenna biramous. Endopod with three articles: article 1 short with five short dorsal setae; article 2 long with two long setae on proximal mid-ventral process, right article 2 with one prominent sharp protrusion on disto-ventral margin; article 3 approximately equilong to article 2, with one long mid-dorsal seta with a list dorsal cilia, two short disto-medial setae, and eight fan-shaped tines on blunt tip, mid-ventral margin hump. Exopod with nine articles: articles 1–8 with a line of fine spines on distal margin; articles 3–8 with one long plumose seta with numerous tiny spines on each disto-dorsal margin; articles 4–8 with one spine on disto-ventral edge; articles 1 and 3 long, others short; article 2 with one grand disto-dorsal seta with several mid-dorsal short spines; and article 9 small with four long plumose setae on tip.

**Mandible (Figs. 1K–1N):** Limb biramous. Basale grand and long; dorsal margin slightly hump shaped with one long dorsal seta and two long distal dorsal setae; ventral margin with four long plumose setae, one short proximo-ventral seta and one long mid-ventral seta, and several long spines on half proximo-ventral part; one short hook-shaped seta and four very short setae on medio-ventral part. Mandible exopod tiny, slender thumb-shaped on disto-dorsal margin of endopod article 2 with short cilia, one short seta and one long plumose seta on tip. Endopod with three articles. Article 1 with five long plumose setae on disto-ventral margin. Article 2 with one short and two long setae with basal articulation on proximo-dorsal margin, one long and one longer setae with basal articulation on mid-dorsal margin, one short and one long setae with basal articulation on disto-ventral margin, three long setae on disto-ventral edge without basal articulation, and three short spines on proximo-ventral margin. Article 3 blunt and short conical with two strong distal knife-shaped claws, two long and thin setae on tip, and two long and thin setae on antero-ventral margin.

**Maxilla (Fig. 2A):** Precoxale and coxale with fringe of cilia along dorsal margin. Coxale with one plumose seta on disto-dorsal edge. Basale with one lateral plumose seta, two disto-dorsal setae and three disto-ventral setae. Exopod with two articles. Article 1 long, with one disto-dorsal seta and three disto-ventral setae. Article 2 very short, with three claws and three long setae on tip. Maxilla with three endites. Endite I with nine plumose setae and two claws. Endite II with three plumose setae and two claws. Endite III with six plumose setae and two claws.

**Fifth limb (Fig. 2B):** Coxale with three endites. Endite I with five plumose setae. Endite II with eight plumose setae. Endite III with 11 plumose setae. Exopod with five articles. Article 1 with two plumose setae on mid-distal margin, main tooth comprising a slice of constituent teeth. Article 2 with three long and two small plumose setae on posterior side. Article 3 with four plumose setae on inner lobe and one slender plumose seta on outer lobe. Articles 4 and 5 fused with seven distal plumose setae.

**Sixth limb (Fig. 2C):** Epipod with three short plumose setae. Endite I with two short and one long plumose setae, middle seta three times length of others. Endite II–IV with four, nine, and eight plumose setae, respectively. Terminal article subtriangle with about 18 plumose setae.

**Seventh limb (Figs. 2D and 2E):** Limb with nine articles. Articles 1 and 3–8 very short and bare. Article 2 short with a long dorsal seta. Article 9 with one long ventral basal seta on comb side and two long antero-dorsal setae on peg side. Setae with two bells each. Comb with four teeth, side opposite comb with single bare bending peg. Inner two comb teeth with two dorsal spines, two ventral spines, one dorsal basal spine, and one ventral basal spine, respectively.

**Furca (Fig. 2F):** Each furcal lamella approximately quadrilateral with slightly inflated base, 10 claws, several unequal ventral cilia, and one small spine on disto-dorsal claw corner. Claws one, two, four, and six primary, long sickle-shaped from long to short in turn arrangement, with several short ventral spines and bare tip, largest main claws with two long medial basal setae. Claws three, five, and seven to 10 secondary, straight, with several short ventral spines and bare base.

**Figure 3:**
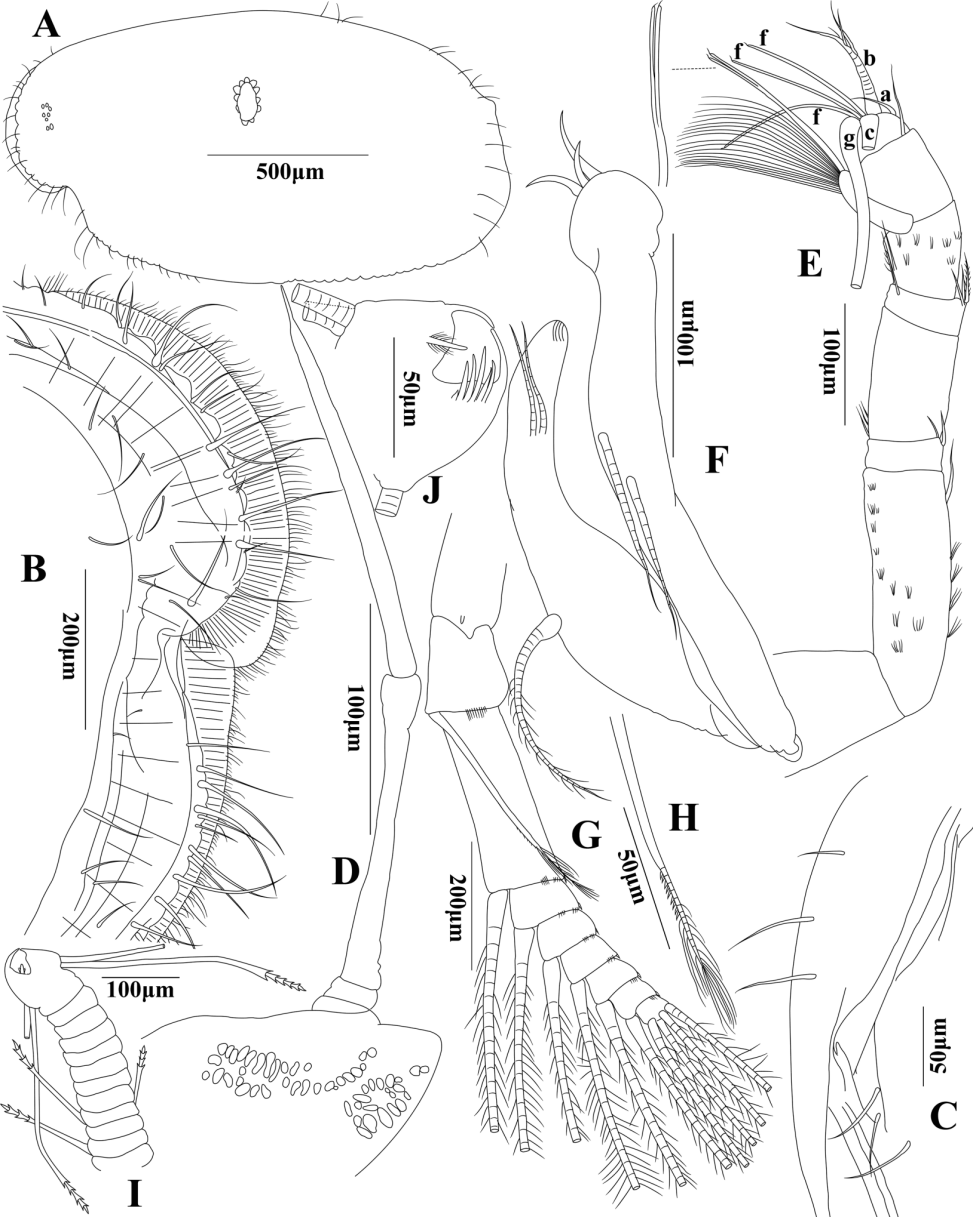
*Euphilomedes pentacanthos* sp. nov. (A) Left carapace, lateral view. (B) Rostrum of left carapace, medial view. (C) Posterior margin of left carapace, medial view. (D) Frontal organ, lateral view. (E) First antenna, lateral view. (F) Endopod of second antenna, lateral view. (G) Exopod of second antenna, lateral view. (H) Seta of exopod two of second antenna, lateral view. (I) Seventh limb, lateral view. (J) Tip of seventh limb, lateral view.

**Figure 4:**
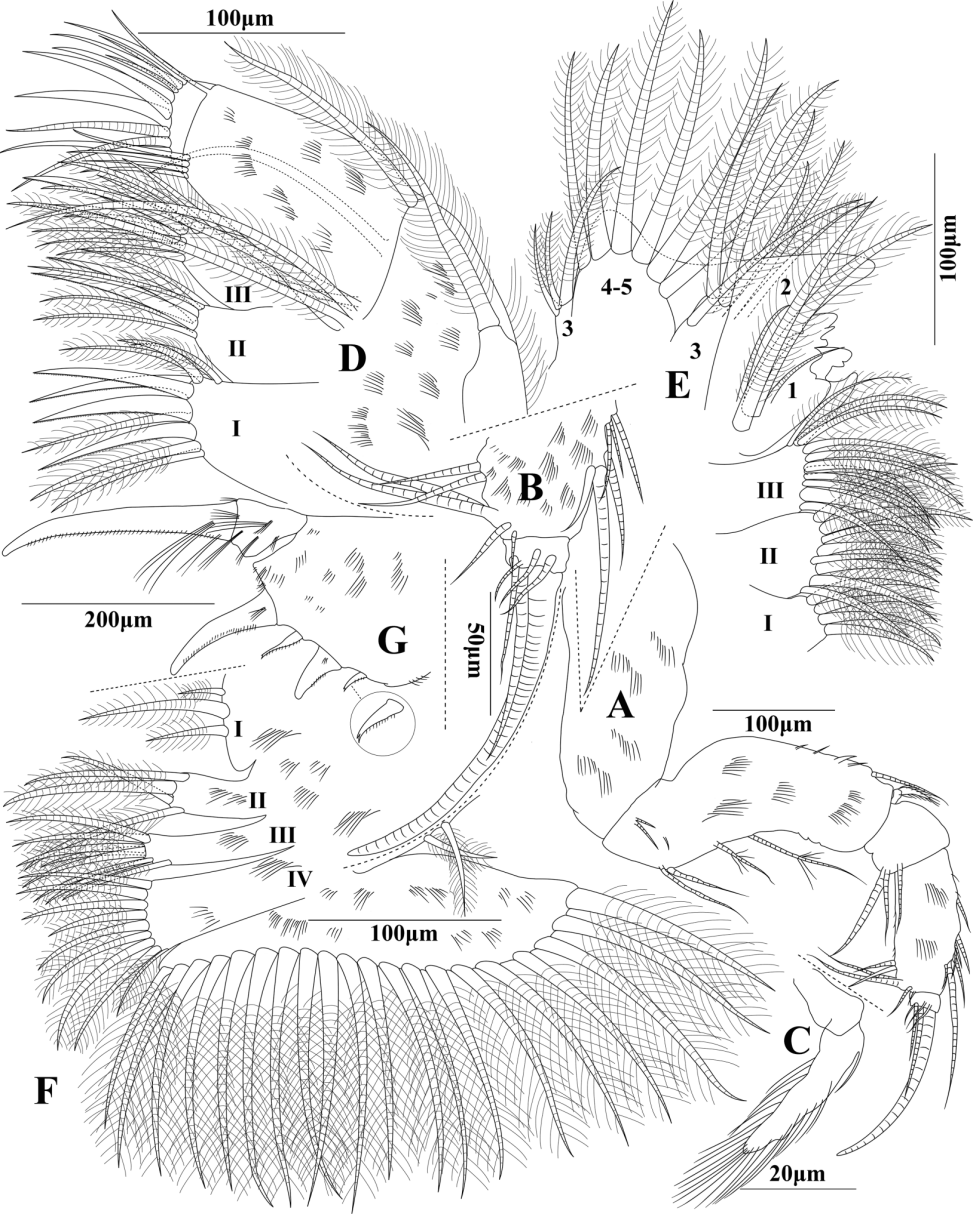
*Euphilomedes pentacanthos* sp. nov. (A) Mandible, lateral view. (B) Tip of mandible, lateral view. (C) Exopod of mandible, lateral view. (D) Maxilla, lateral view. (E) Fifth limb, lateral view. (F) Sixth limb, lateral view. (G) Furca, lateral view.

***Euphilomedes pentacanthos* sp. nov. Chen & Xiang**
urn:lsid:zoobank.org:act:5221A51B-C495-4324-85ED-0BDA00E77D0B
Figures 3 and 4.

**Etymology:** *Pentacanthos*, derived from Latin expression of five claws, indicates that this species have five furcal claws.

**Holotype:** Collection number TIO-OMPEu 311, adult male, length 1.54 mm, height 0.85 mm, collected from the west coast of the Taiwan Strait (24°16.50′N, 118°53.00′E; depth 36 m), Feb 27, 1985.

**Paratypes:** Collection number TIO-OMPEu 312, adult male, length 1.50 mm, height 0.82 mm, dissected on slides, carapace in alcohol. Paratype collected in the same way as the holotype.

**Distribution:** Known at present only from type locality.

**Diagnosis:** Posterior margin of carapace approximately flat, forming a right angle with dorsal carapace margin. Mandibular exopod with two articles, other species of *Euphilomedes* mandibular exopod with only one article. Comb of seventh limb with six long teeth and one very short inner tooth, side opposite comb with one outer spindly and bending peg and one inner long plumose spine with five short dorsal cilia and five short ventral cilia. Each furcal lamella with only five furcal claws, claws one, two, and four primary; this species has minimum number of furcal claws of all known *Euphilomedes* species.

### Description

**Carapace (Figs. 3A–3C):** Carapace ornamentation: carapace oval in lateral view with small round and oval carapace protrusions on surface and long setae on posterior margin, projecting rostrum and shallow incisure, rostral infold depressed, the list of rostrum and ventral margin with several unequal setae. Rostrum and ventral margin with continuous small slightly arches. Posterior carapace margin approximately flat, approximately forming a right angle with dorsal carapace margin. Carapace size: length 1.54 mm, height 0.85 mm, height about 55% of length.

**First antenna (Fig. 3D):** First antenna uniramous with eight articles. Article 1 grand and bare. Article 2 very long approximately two times length of article 4 with several clusters of small spines and one disto-dorsal long seta. Article 3 very short approximately one-third length of article 4 with two short setae on disto-dorsal edge. Article 4 long with three short setae on ventral basal margin. Article 5 very short equilong to article 3 with two short setae and one short plumose seta on disto-dorsal edge with basal articulation, and one short plumose seta on disto-ventral margin. Article 6 short approximately two times length of article 5 with several clusters of small spines, one proximo-ventral straight seta, and one big seta approximately equilong to article 6 on disto-ventral edge with about 20 very long soft disto-vental filaments and one very long distal bifurcate seta with a hook-shaped cilium on tip, respectively. Article 7 equilong to article 6 with a long seta on disto-dorsal edge. Article 8 blunt and short conical with seven sensory setae on tip, a-seta short, b-seta stick and strong with three long spines on disto-dorsal margin, c- and g-setae grand and very long, three f-setae long and thin with several short cilia and a hook-shaped cilium on tip, respectively, without d- and e-setae.

**Frontal organ (Fig. 3E):** Frontal organ extremely long and thin with three articles. Article 1 extremely short; articles 2 and 3 long; and article 3: with a narrow tip.

**Second antenna (Figs. 3F–3H):** Second antenna biramous. Endopod with three articles: article 1 very short with three stick-shaped and short ventral setae; article 2 with two long setae on mid-ventral margin; and article 3 with a corpulent proximal half part, one long mid-dorsal plumose seta, two long equilong medio-distal setae, and a blunt tip with four microgrooves. Exopod with nine articles: article 1 long with one small medio-distal digitation and one big medio-distal marginal digitation; articles 2–8 with one long plumose seta and small cilia on disto-dorsal edge, respectively; plumose seta on article 2 with small spines on mid-ventral margin; article 3 long; articles 3–8 with one spine on disto-dorsal edge; article 9 very small with four long plumose setae on tip.

**Mandible (Figs. 4A–4C):** Limb biramous. Coxale long grand and bare. Basale long approximately equilong to coxale, with one long disto-dorsal seta with basal articulation, four short disto-dorsal spines, three short medial proximal spines, two long proximo-ventral equilong setae, and two long mid-ventral plumose setae. Exopod small on disto-dorsal edge of basale with two articles: article 1 tiny and bare, article 2 with one long dorsal basal spine and several long plumose filaments. Endopod with three articles: article 1 very short with three long setae on disto-ventral edge; article 2 approximately two times length of article 1, with several clusters of small spines, two short proximo-dorsal spines, one short dorsal seta with basal articulation, one cluster of three short mid-dorsal setae with basal articulation, one cluster of three long disto-dorsal setae with basal articulation, two short mid-ventral spines, two long disto-ventral setae, two very long and two short disto-ventral marginal setae; and article 3 blunt and short conical, with one grand and one small sword-shaped seta on tip, and four short setae on medial distal margin.

**Maxilla (Fig. 4D):** Precoxale and coxale with fringe of cilia along dorsal margin. Coxale with one plumose seta on disto-dorsal edge. Basale with one lateral plumose seta, two disto-dorsal setae and five disto-ventral setae. Exopod with two articles. Article 1 long and broad, with one disto-dorsal seta and four disto-ventral setae. Article 2 extremely short, with three claws, two small setae, two big claws, and three long setae on tip. Endite I with six plumose setae and four claws. Endite II with four plumose setae and two claws. Endite III with eight plumose setae and two claws.

**Fifth limb (Fig. 4E):** Endites I–III of coxale with four, eight, and 11 plumose setae, respectively. Exopod one: with two plumose setae on distal edge, main tooth comprising some constituent teeth. Exopod two: with two long and two small plumose setae on posterior side, middle setae approximately two times length of ambilateral setae. Exopod three: with three plumose setae on inner lobe, one long and two short plumose setae on outer lobe. Exopod four and five: fused, with six plumose setae on tip.

**Sixth limb (Fig. 4F):** Limb with clusters of small cilia on board surface. Epipod with two short plumose setae. Endite I: with three irregular plumose setae, middle seta longest. Endite II with four plumose setae. Endite III with eight plumose setae. Endite III with eight long and one small plumose setae. Terminal article compressed with about 20 long plumose setae.

**Seventh limb (Figs. 3I and 3J):** Limb with 13 articles. Articles one to 11: very short with terminal fused articles 12 and 13. Articles 1, 3, and 5–11 bare. Article 2 with one short ventral seta. Article 4 with one short ventral and one short dorsal setae. Ventral basal edge and dorsal basal edge of article 13 with two very long setae, respectively. Comb with six long teeth and one very short inner tooth, side opposite comb with one outer gangly and bending peg and one inner long plumose spine with five short dorsal cilia and five short ventral cilia. Setae on terminal article with five bells each, other setae with three bells each.

**Furca (Fig. 4F):** Each furcal lamella with five furcal claws and several clusters of spines. Claws one, two, and four primary, long sickle-shaped from long to short in turn arrangement, with several short ventral spines and bare blunt tips. Claw one with long basal spines, claws two and four with short basal spines. Claw one with a big trigonal end joint. Claws three and five secondary, with several small ventral spines and a small sharp bifurcate tip.

## Discussion

With the two new species described in this study, the genus *Euphilomedes* now contains 31 recent species (Brandão et al., 2016), including 10 species recorded in Chinese waters, such as *Euphilomedes corrugate*, *Euphilomedes interpuncta*, *Euphilomedes japonicus*, *Euphilomedes longiseta*, *Euphilomedes multiangular*, *Euphilomedes nodosa*, *Euphilomedes sordida, Euphilomedes spinulosa* and these two new species (Tseng, 1970; Chen & Lin, 1995, 1997; Liu, 2008; Chen, 2012; Chen et al., 2015a, 2015b). However, it is worth noting that more new species await exact descriptions in further investigations.

In addition to the shape of the carapace, first antenna, mandible, second antenna and frontal organ, the structure of terminal article of the seventh limb, the number of furcal claws and arrangement between the main claws and secondary claws are also the key characteristics in species identification within *Euphilomedes*. Both *Euphilomedes liuruiyii* sp. nov. and *Euphilomedes pentacanthos* sp. nov. are most closely related to *Euphilomedes asper* (Müller, 1894) collected from the Bay of Naples between depths from 52 to 187 m (Müller, 1894; Poulsen, 1962). They have several shared characteristics as below that separate them from the other *Euphilomedes* species: (1) the carapace has pits and no cluster of spines on ventral margin (Figs. 1A and 3A); (2) the rostrum has evenly rounded margin and the incisure is shallow (Figs. 1B and 3B); (3) the postero-ventral corner has no sharp bulge; (4) the carapace valve has no spines on posterior end; (5) there is no process on postero-dorsal margin of the carapace; (6) the endopod three of first antenna bears distal setae (Figs. 1C and 3E); (7) the setae on endopod three of the second antenna endopod are normally (spinose and ringed) for male individual (Figs. 1F, 1H and 3F); (8) they have similar structures of maxilla, fifth limb, and sixth limb (Figs. 2A–2C and 4D–4F); (9) side opposite comb of seventh limb has peg(s) (Figs. 2E and 3J); (10) furcal lamella has secondary claws after the dorsal main claw (Figs. 2F and 4G); and (11) the fourth furcal claw is main claw.

With more detailed observations, the two new species in this study were distinguished from *Euphilomedes asper* based on their morphological comparisons (Tables 1 and 2). Although they also differ in the number of cleaning setae on the seventh limb, this characteristic is variable (Chavtur & Angel, 2011), therefore, we did not include this characteristic for comparisons. The identifications and separations of the two new species from *Euphilomedes asper* are described below:

(A) *Euphilomedes liuruiyii* sp. nov.: (1) rostrum is projecting, incisure is shallow, rostral infold is depressed and bears distal and ventral cilia (Fig. 1B); (2) endopod three of the second antenna has one long mid-dorsal seta with a list of dorsal cilia and eight fan-shaped tines on the blunt tip, mid-ventral margin is hump (Figs. 1F–1I); (3) mandibular basale bears one short hook-shaped seta on proximo-ventral part (Figs. 1L and 1M); (4) comb of the seventh limb has four teeth, and there are two basal spines on each of two inner comb teeth, side opposite comb has one bare bending peg (Fig. 2E); (5) each furcal lamella has 10 claws, clusters of spines, and one small spine on disto-dorsal claw corner (Fig. 2E); (6) claws one, two, four, and six are main claws, claws three, five, and seven to 10 are secondary claws (Fig. 2F); and (7) main claws have basal spines clusters, claw one has the longest spines clusters (Fig. 2G).

(B) *Euphilomedes pentacanthos* sp. nov.: (1) has a big arced hump on carapace postero-ventral margin, and continuous small slightly arches on the rostrum and ventral margin, and the posterior carapace margin is approximately flat (Fig. 3A); (2) has no vertical distal cilia on article 8 of the first antenna (Fig. 3E); (3) frontal organ is extremely long and thin with three articles, and the article 3 has a narrow tip (Fig. 3D); (4) has one long mid-dorsal plumose seta and two long medio-distal setae on endopod three of second antenna (Fig. 3F); (5) comb of the seventh limb has six long teeth and one extremely short inner tooth (Fig. 3J); (6) side opposite comb has one outer spindly and bending peg, and one inner long plumose spine with five dorsal and five ventral short cilia; and (7) each furcal lamella has five furcal claws, claws one, two, and four are main claws; claws three and five are secondary claws with several small ventral spines and small sharp bifurcate tip (Fig. 4G).

**Table 1:** Comparisons between *E. liuruiyii* sp. nov. and *E. asper* (Müller, 1894).

Characteristics	*E. liuruiyii* sp. nov. (♂)	*E. asper* (♂)
Carapace	Projecting rostrum and shallow incisure, rostral infold depressed with distal cilia and ventral cilia	Obviously projecting rostrum and clearer incisure
Endopod of second antenna	Article 3 with one long mid-dorsal seta with a list dorsal cilia, and eight fan-shaped tines on blunt tip, mid-ventral margin hump	Article 3 with one very short proximo-dorsal seta, and a blunt tip with some microgrooves, without ventral margin hump
Mandible	Basale with one short hook-shaped seta on medial–proximal–ventral part	No hook-shaped seta on basale
Seventh limb	Comb with four teeth, two inner comb teeth with two basal spines each; side opposite comb with one single bare bending peg	Comb with six tooth with five small basal spines, side opposite comb with one long and one short ventral comb pegs
Furca	Each furcal lamella with 10 claws, clusters of spines, and one small spine on disto-dorsal claw corner Claw one, two, four and six main claws; claw three, five, and seven to 10 secondary claws Main claws with basal spines clusters, claw one with longest spines clusters	Each furcal lamella with 10 claws, no small spine on distal-dorsal claw corner Claw one, two, four main claws; claw three, and five to 10 secondary claws

**Table 2:** Comparisons between *E. pentacanthos* sp. nov. and *E. asper* (Müller, 1894).

Characteristics	*E. pentacanthos* sp. nov. (♂)	*E. asper* (♂)
Carapace	Postero-ventral margin of carapace big circular arc hump, posterior carapace margin approximately flat, rostrum and ventral margin with continuous small slightly circular arc	Postero-ventral margin of carapace slightly circular arc hump, posterior carapace margin arc shaped, no continuous small slightly circular arc on rostrum margin
First antenna	No vertical distal cilia on article 8	Article 8 with two lists of vertical distal cilia
Frontal organ	Frontal organ extremely long and thin with three articles, article 3 with a narrow tip	Frontal organ with two articles, article 2 with a lanceolar tip
Endopod of second antenna	Article 3 with one long mid-dorsal plumose seta, two long medial-distal setae	Article 3 with only one short seta
Seventh limb	Comb with six long teeth and one very short inner tooth, side opposite comb with one outer long thin and bending peg and one inner long plumose spine with five short dorsal cilia and five short ventral cilia	Comb with six tooth, small spines inserted between teeth, side opposite comb with two long pegs
Furca	Each furcal lamella with five furcal claws Claw one, two, and four main claws; claw three and five secondary claw, with several small ventral spines, and a small sharp bifurcate tip	Each furcal lamella with 10 claws Claw one, two, four main claws; claw three, and five to 10 secondary claws

Remarkably, for *Euphilomedes liuruiyii* sp. nov., there is a prominent sharp protrusion on the disto-ventral margin of right endopod two of the second antenna (Fig. 1H), a distinctive feature not previously observed in genus *Euphilomedes*. In terms of *Euphilomedes pentacanthos* sp. nov., it has only five furcal claws, which is the lowest recorded number of the furcal claws of species in *Euphilomedes*.

In the future, more intensive studies based on morphologic descriptions and molecular analyses are warranted to obtain a better understanding of the diversity and evolution of this genus.

## Supplemental Information

10.7717/peerj.3146/supp-1 Supplemental Information 1 List of ostracod species of genus *Euphilomedes* Poulsen, 1962.

## Additional Information and Declarations

### Data Availability

The following information was supplied regarding data availability: The raw data is included in the manuscript in figures and Results section.

### New Species Registration

The following information was supplied regarding the registration of a newly described species: *Euphilomedes liuruiyii* sp. nov.: urn:lsid:zoobank.org:act:51A26454-F244-4C42-921A-667846DF529D. *Euphilomedes pentacanthos* sp. nov.: urn:lsid:zoobank.org:act:5221A51B-C495-4324-85ED-0BDA00E77D0B. The LSID for this publication is: urn:lsid:zoobank.org:pub:9E34993C-1881-4D7F-919B-BCA046F411BB.
