# Supplementary material for: Two new benthic Euphilomedes Kornicker, 1967 (Ostracoda, Myodocopida, Philomedidae) from the Taiwan Strait (East China Sea)
Source: PeerJ. 2017 Apr 6;5:e3146. doi: 10.7717/peerj.3146 (PMC5385131; doi:10.7717/peerj.3146)
Supplement: Supplemental Information 1 [file peerj-05-3146-s001.doc]

**Supplemental Table 1** List of ostracod species of genus *Euphilomedes* Poulsen, 1962.

**Extracted from WOD (Brandão et al., 2016).**

| **Species** | **Type locality** | **Reference(s)** |
| --- | --- | --- |
| ***E. interpuncta* (Baird, 1850)** | Irish coastal waters | **Baird, 1850; Poulsen, 1962; Brandão et al., 2016** |
| ***E. japonicus* (Müller, 1890)** | Japanese coastal waters, depth 18.3-36.6 m. | **Müller, 1890; Poulsen, 1962** |
| ***E. sordidus* (Müller, 1890)** | Japanese coastal waters, depth 0-4 m. | **Müller, 1890; Poulsen, 1962; Brandão et al., 2016** |
| ***E. moroides* (Brady, 1890)** | South Sea Islands of Australia, between tide-marks, fringing reefs, to depth 11 m. | **Brady, 1890; Poulsen, 1962** |
| ***E. asper* (Müller, 1894)** | Bay of Naples, depth 52-187 m. | **Müller, 1894; Poulsen, 1962; Brandão et al., 2016** |
| ***E. corrugata* (Brady, 1897)** | Bay of Naples, depth 5.5-7.3 m. | **Brady, 1897; Poulsen, 1962; Brandão et al., 2016** |
| ***E. debilis* (Brady, 1902)** | Off Trinconali of Ceylon. | **Brady, 1902; Poulsen, 1962** |
| ***E. longiseta* (Juday, 1907)** | West coast of North America, depth 5.5-7.3 m. | **Juday, 1907; Poulsen, 1962; Brandão et al., 2016** |
| ***E. ijimai* (Kajiyama, 1912)** | Shallow water of Mikawa Bays, Pacific coast of central Japan. | **Kajiyama, 1912;**  **Hiruta, 1976** |
| ***E. africana* (Klie, 1940)** | Shallow waters off west coast of South Africa. | **Klie, 1940; Poulsen, 1962; Brandão et al., 2016** |
| ***E. carcharodonta* (Smith, 1952)** | Pacific coast of Canada depth 5.5-7.3 m. | **Smith, 1952; Poulsen, 1962** |
| ***E. nodosa* Poulsen, 1962** | Off southern Thailand, depth 2 m. | **Poulsen, 1962; Brandão et al., 2016** |
| ***E. bradyi* Poulsen, 1962** | Red Sea (males collected from surface water at night). | **Poulsen, 1962** |
| ***E. smithi* Poulsen, 1962** | San Jose gulf waters of Panama Gulf, depth 9 m. | **Poulsen, 1962** |
| ***E. producta* Poulsen, 1962** | West coast of North America, depth 13-401 m | **Poulsen, 1962; Brandão et al., 2016** |
| ***E. walfordi* Poulsen, 1962** | Coral Sea, depth 50 m. | **Poulsen, 1962** |
| ***E. sinister* Kornicker, 1974** | Bay of Naples, depth 90 m. | **Kornicker, 1974; Brandão et al., 2016** |
| ***E. kornickeri* Hartmann, 1974** | Coastal waters of Africa. | **Hartmann, 1974; Brandão et al., 2016** |
| ***E. nipponicus* Hiruta, 1976** | Japanese Vicinity waters, depth 4 m. | **Hiruta, 1976; Brandão et al., 2016** |
| ***E. caudata* Hartmann, 1985** | Offshore of Ibérian Peninsula. | **Hartmann, 1985** |
| ***E. clima*x Kornicker, 1991** | Eastern Pacific, depth 1700 m. | **Kornicker, 1991; Brandão et al., 2016** |
| ***E. ernyx* Kornicker, 1995** | Eden inshore of New South Wales, depth 220 m. | **Kornicker, 1995. ; Brandão et al., 2016** |
| ***E. morini* Kornicker & Harrison-Nelson, 1997** | Pillar Point Harbor, Half Moon Bay, California. | **Kornicker & Harrison-Nelson, 1997; Brandão et al., 2016** |
| ***E. cooki* Harrison-Nelson & Kornicker, 2000** | Moreton Bay of Queensland, depth 15-25 m. | **Harrison-Nelson & Kornicker, 2000; Brandão et al., 2016** |
| ***E. pseudosordidus* Chavtur, Shornikov, Lee & Huh, 2007** | Shallow waters off the west coast of South Africa. | **Chavtur *et al*., 2007; Brandão et al., 2016** |
| ***E. chupacabra* Lum, Syme, Schwab & Oakley, 2008** | Along the Puerto Rico coast, depth 2-20 m. | **Lum *et al.*, 2008; Brandão et al., 2016** |
| ***E. tasmanicus* Karanovic, 2010** | Off Tasmania, Australia, depth 13 m. | **Karanovic, 2010; Brandão et al., 2016** |
| ***E. multiangular* Chen & Xiang, 2015** | Coastal waters of Western Taiwan Strait, China, depth 67m. | **Chen *et al*., 2015** |
| ***E. spinulosa* Chen, Xiang & Chen, 2015** | **Bay of Dongshan, China, depth 17 m.** | **Chen *et al*., 2015** |

**REFERENCES**

**Baird W. 1850.** Description of several new species of Entomostraca. *Proceedings of the Zoological Society of London* 18:254–257.

**Brady GS. 1890.** On Ostracoda collected by H.B. Brady, Esq., L.L.D., F.R.S., in the South Sea Islands. *Transactions of the Royal Society of Edinburgh: Earth Sciences* 35:489–525.

**Brady GS. 1897.** A supplementary report on the Crustaceans of the group Myodocopa obtained during the “Challenger” Expedition, with notes on other new or imperfectly known species. *Transactions of the Zoological Society of London* 14:85–100.

**Brady GS. 1902.** On new or imperfectly known Ostracoda, chiefly from a Collection in the Zoological Museum, Copenhagen. *Transactions of the Zoological Society of London* 16:179–210.

**Brandão, SN, Angel MV, Karanovic I, Parker A, Perrier V & Yasuhara M.** 2016. *World Ostracoda Database*. Accessed at http://www.marinespecies.org/Ostracoda on 2017-01-03.

**Chavtur VG, Shornikov EI, Lee E-H, Huh M. 2007.** Benthic Ostracoda (Myodocopina, Philomedidae) of the East Sea (Sea of Japan), with description of a new species from the Korean Peninsula. *Zootaxa* 1531:1–24.

**Chen X, Xiang P, Chen R, Lin M. 2015.** A new species of *Euphilomedes* from the Taiwan Strait, (Ostracoda: Philomedidae). *Journal of Fisheries of China* 39(4): 505–510.

**Chen X, Xiang P, Chen R, Lin J, Lin M. 2015.** A new species of *Euphilomedes* from the Dongshan Strait (Ostracoda: Myodocopida: Cypridinacea). *Acta Oceanologica Sinica* 37(8): 126–133.

**Harrison-Nelson EM, Kornicker LS. 2000.** *Euphilomedes cooki*, a new species of myodocopid ostracode from Mereton Bay, SE Queensland, Australia. *Proceedings of the Biological Society of Washington* 113(2):465–749.

**Hartmann-Schröder G, Hartmann G. 1974.** Zur Kenntnis des Eulitorals der afrikanischen Westküeste zwischen Angola und Kap der Guten Hoffnung und der afrikanischen Ostküeste von Süedafrika und Moçambique unter besonderer Berüecksichtigung der Polychaeten und Ostracoden Teil 3: Die Ostracoden des Unte G. Hartmann-Schroeder and Hartmann G, eds. *Mitteilungen aus dem Hamburgischen Zoologischen Museum und Institut 69 (Ergaenz.)*, 229–521.

**Hartmann G. 1985.** Ostracoden aus der Tiefsee des Indischen Ozeans und der Iberischen See sowie von ostatlantischen sublitoralen Plateaus und Kuppen. *Mit einer Tabelle der bislang bekannten rezenten Tiefseeostracoden. Senckenb Marit* 17:89–146.

**Hiruta S. 1976.** *Euphilomedes nipponica* n. sp. from Hokkaido, with a Redescription of *E. sordida* (GW. Müller) (Ostracoda; Myodocopina) (With 12 Text-figures). *Journal of the Faculty of Science Hokkaido University Series VI, Zoology* 20(3):579–599.

**Juday C. 1907.** Ostracoda of the San Diego region. II: Littoral forms. *Univ. California Publ. Zool* 3:135–156.

**Kajiyama E.** **1912.** On the Ostracoda of Misaki (part 2). *Zoological Magazine (Dobutugaku-zasshi)* 25:609–619.

**Karanovic I. 2010.** A new *Euphilomedes* Kornicker, 1967 (Myodocopida: Philomedidae) from Tasmania with a key to the species of the genus. *Marine Biodiversity* 40(3):219–236.

**Klie W. 1940.** Beiträge zur Fauna des Eulitorals von Deutsch-Südwest-Afrika. II. Ostracoden von der Küste Deutsch-Süedwest-Afrikas. *Kieler Meeresforschungen* 3:403–448.

**Kornicker LS. 1974.** Revision of the Cypridinacea of the Gulf of Naples (Ostracoda). *Smithsonian Contributions to Zoology* 178:1–60.

**Kornicker LS. 1991.** Myodocopid Ostracoda of hydrothermal vents in the eastern Pacific Ocean. *Smithsonian Contributions to Zoology* 516:1–46.

**Kornicker LS. 1995.** Ostracoda (Myodocopina) of the SE Australian Continental Slope, Part 2. *Smithsonian Contributions to Zoology* 562:1–97.

**Kornicker LS, Harrison-Nelson EM. 1997.** Myodocopid Ostracoda of Pillar Point Harbor, Half Moon Bay, California. *Smithsonian Contributions to Zoology* 593:1–53.

**Lum KE, Syme AE, Schwab AK, Oakley TH. 2008.** *Euphilomedes chupacabra* (Ostracoda: Myodocopida: Philomedidae), a new demersal marine species from coastal Puerto Rico with male-biased vespertine swimming activity. *Zootaxa* 1684:35–57.

**Müller GW. 1890.** Neue Cypridiniden. *Zoologische Jahrbüecher Abteilung für Systematik Geographie und Biologie der Thiere* 5:211–252.

**Müller GW. 1894.** Die Ostracoden des Golfes von Neapel und der angrenzenden Meeres-Abschnirte. In: Neapel ZS zu, eds. *Fauna und Flora Golf von Neapel und der angrenzenden Meeres-Abschnitte*. Berlin, 1–404.

**Poulsen EM. 1962.** Ostracoda-Myodocopa, Part I: Cyridiniformes-Cypridinidae. *Dana Report* 57:1–414.

**Smith VZ. 1952.** Further Ostracoda of the Vancouver Island Region. *Journal of the Fisheries Research Board of Canada* 9:16–41.
